# Functional identification of the calcineurin B-like protein PavCBL4 in modulating salt tolerance in sweet cherry

**DOI:** 10.3389/fpls.2023.1293167

**Published:** 2023-11-22

**Authors:** Quanjuan Fu, Sen Hou, Rui Gao, Guoqin Wei, Yugang Sun

**Affiliations:** Shandong Institution of Pomology, Taian, Shandong, China

**Keywords:** sweet cherry (*Prunus avium*), calcineurin B-like proteins (CBLs), salt stress, PavCBL4, Na^+^ accumulation, antioxidant enzyme

## Abstract

Abiotic stresses, such as high salinity, pose a significant threat to plant growth and development, reducing crop yield and quality. Calcineurin B-like (CBL) proteins serve as crucial calcium sensors in plant responses to diverse environmental stresses. However, the CBL family in sweet cherry has not been identified at the genome-wide level, and the regulatory role of CBL proteins in cherry plants’ salt response is unclear. Here, we identified 10 CBL family genes (*PavCBLs*) from the *Prunus avium* genome and cloned seven of them. We comprehensively analyzed *PavCBL* genes for collinearity, phylogenetic relationships, gene structure, and conserved motifs. Expression analysis revealed significant induction of transcription under abiotic stress, with *PavCBL4* displaying the most substantial expression change. Additionally, we identified PavCBL4 as a PavSOS2 (Salt Overly Sensitive 2)-interacting protein through Y2H and Split-LUC assays. Subcellular localization analysis indicated that PavCBL4 is present in both the cytoplasm and nucleus. Functional assessment of *PavCBL4* in the *PavCBL4*-overexpressing transgenic ‘Gisela 6’ plants showed its positive role in enhancing salt tolerance in cherry plants. Measurements of Na^+^ content and antioxidant enzyme activity under salt stress indicated that *PavCBL4* functions positively by inhibiting Na^+^ accumulation and promoting ROS scavenging in response to salt stress. These findings lay the groundwork for a deeper understanding of the molecular mechanisms underlying *PavCBL*-mediated salt tolerance in sweet cherry.

## Introduction

Plants, immobile by nature, must adapt to environmental changes like pathogens, drought, soil salinity, and extreme temperatures (Dong et al., 2022), significantly affecting global crop growth and productivity. Detecting and interpreting stimuli is crucial for plants to trigger suitable survival responses in challenging settings (Kissoudis et al., 2014). Ca^2+^ plays a pivotal role in various plant processes: growth, development, and biotic and abiotic stress responses (Harmon, 2003). The Ca^2+^ signal conveys information about stimuli, activating specific molecular and physiological responses and serving as a crucial specificity layer (Poovaiah and Du, 2018). Additionally, Ca^2+^-binding proteins and their interactors provide further specificity (Aldon et al., 2018), collectively constituting classical calcium signaling.

In plants, the Ca^2+^ signals are detected by different types of Ca^2+^-binding sensors. These include calmodulin (CaM), CaM-like (CML) proteins, calcineurin B-like (CBL) proteins, Ca^2+^-dependent protein kinases (CDPKs), and calcium/CaM-dependent protein kinase (CCaMK) (Poovaiah and Du, 2018). The CBL family, resembling the calcineurin B subunit of yeast and animal cell protein phosphatases, possesses four elongation factor (EF)-hand motifs that can bind up to four Ca^2+^ molecules (Kudla et al., 1999; Nagae et al., 2003; Sanchez-Barrena et al., 2005). Studies across plant species highlight CBLs’ significant role in responding to adverse environments. In *Arabidopsis*, 10 CBL proteins, grouped based on their phylogenetic relationship, subcellular localization, and function, have been identified (Kolukisaoglu et al., 2004). The CBL family’s characterization extends to various plant species, including *Populus euphratica* (Zhang et al., 2008), *Vitis vinifera* (Xi et al., 2017), and *Solanum melongena* (Li et al., 2016).

CBLs, proteins interacting with CIPKs (CBL-interacting protein kinases) (Yadav et al., 2018), translate signals into phosphorylation events, activating plant responses to environmental stresses like high salinity (Albrecht et al., 2001; Xuan et al., 2019). Recent studies have identified many CBL-CIPK signal components that are crucial in abiotic stress signaling pathways (Albrecht et al., 2003; Gao et al., 2020). The CBL-CIPK complex regulates Na^+^ accumulation and K^+^ levels, crucial for plant salt tolerance. For instance, CBL1/9-CIPK23 enhances K^+^ uptake, activating AKT1 during K^+^ deficiency (Xu et al., 2006; Cheong et al., 2007). This complex also reportedly affects cassava’s low potassium stress response (Yan et al., 2021). Additionally, CBL3-CIPK9 may regulate cellular K^+^ homeostasis under potassium deficiency by controlling K^+^ transport across the vacuolar membrane (Amtmann and Armengaud, 2007; Liu et al., 2013). Moreover, CBL1/CBL9-CIPK23 complex regulate nitrate (NO_3_. ^−^) sensing and uptake via regulating the nitrate sensor and transporter CHL1 (Ho et al., 2009).

Salt stress significantly impacts plant growth and crop productivity (Yang and Guo, 2018). Studies have shown that CBLs regulate plant adaptation to salt stress via a CIPK-dependent salt overly sensitive (SOS) pathway. This pathway facilitates Na^+^ ion transport out of the cell, which confers plant salt stress tolerance (Liu and Zhu, 1998; Yang and Guo, 2018). In general, SOS3 (CBL4) interacts with SOS2 (CIPK24) in response to salt stress to jointly activate the transmembrane transporter SOS1 (NHX7), thus promoting Na^+^ efflux and consequently enhances plant salt tolerance (Liu and Zhu, 1998; Qiu et al., 2002). Recent studies showed that the CBL-CIPK complex regulates transport of other ions. For instance, SCaBP3/CBL3 modulates plasma membrane H^+^-ATPase activity in *Arabidopsis* (Yang et al., 2019). In *Arabidopsis*, NRAMP1, a plasma membrane transporter, primarily mediates Mn absorption (Cailliatte et al., 2010). The CBL1/9-CIPK23 complex helps to avoid manganese toxicity by converting the Ca^2+^ signature into a phosphorylation-mediated regulation of NRAMP1 (Zhang et al., 2023). Furthermore, CPK5 and CBL2/3-CIPK3/9/26 sequentially phosphorylate tonoplast-localized Mn transporter MTP8, providing a regulatory mechanism to fine-tune transporter activation in response to Mn toxicity (Ju et al., 2022).

Sweet cherry (*Prunus avium*), a Rosaceae family fruit tree common in temperate regions, faces challenges such as high salinity, impacting fruit yield and quality (Esti et al., 2002; Shen et al., 2021). Adverse environmental conditions, including high salinity, severely limit cherry fruit yield and quality. However, the CBL family (*PavCBL*) in sweet cherry and its role in regulating salinity tolerance remains uncharacterized and limited. This research involved genome-wide identification and cloning of *CBL* family genes in sweet cherry. Various bioinformatic analyses detailed the identified *PavCBLs*. Furthermore, the study explored *PavCBL* gene expression changes under salt stress and interactions between PavCBLs and PavSOS2. Moreover, the role of *PavCBL4* in regulating cherry salt tolerance was identified in deatil using transgenic ‘Gisela 6’ plants. The findings elucidated the mechanistic understanding of *PavCBL4*-mediated salt tolerance, highlighting its role in inhibiting Na^+^ accumulation and promoting ROS scavenging.

## Materials and methods

### CBL family protein sequence retrieval and identification in cherry

The sweet cherry proteome file was downloaded form the GDR database (https://www.rosaceae.org/; *Prunus avium* Tieton Genome v2.0) (Wang et al., 2020a). The TAIR database (https://www.org/) provided the protein sequences for 10 *Arabidopsis* CBLs (AtCBLs). The Pfam database (http://pfam.xfam.org/) was used to retrieve the HMM file EF-hand_7.hmm (PF13499.8). It was then used as a query to search the sweet cherry proteome by HMMER software (version 3.1b2; http://hmmer.org/), along with a E-value threshold of < 0.01. To search for potential PavCBLs, the protein sequences in HMMER screening results and the 10 AtCBLs were used for phylogenetic analysis (**Figure S1**).

### Bioinformatics analysis of *PavCBLs* gene sequences

The GDR database provided the GFF file (Prunus_avium_Tieton.annotation.gff3) containing *PavCBL* genes location data. The collinearity analysis was conducted using the MCScanX software, with visualization using TBtools (Chen et al., 2020). Phylogenetic trees were generated through MEGAX software (version 10.05) employing the NJ (Neighbor-Joining) method. Gene structures’ schematic diagrams were drawn using GSDS software (version 2.0; http://gsds.gao-lab.org/). Conserved motifs within PavCBLs protein sequences were identified via MEME (version 5.5.2; https://meme-suite.org/meme/tools/meme). The PavCBLs and AtCBLs protein sequences’ alignment was performed using DNAMAN software (version 6). For promoter analysis, 2000 bp-long sequences upstream of the start codon of *Prunus avium PavCBL* genes were collected and analyzed using the online tool PlantCARE (http://bioinformatics.ugent.be/webtools/plantcare/html/). Detailed methods can be found in previous studies (Mao et al., 2017; Mao et al., 2021).

### Plant materials, growth conditions, and salt stress treatment

Tissue-cultured ‘Gisela 6’ sweet cherry rootstock seedlings were subjected to abiotic stress treatments to analyze *PavCBL* gene expression patterns. The seedlings were grown in 1/2 MS rooting medium with 1.0 mg/L IBA, 3% sucrose (w/v), and 0.6% Bacto-agar under long-day conditions (16 h light/8 h dark) at 25 °C. After 35 days, they were transplanted into pots with a 1:1 ratio of nutrient soil and perlite. Following a month, plantlets were chosen and cultured under hydroponic conditions for stress treatments: NaCl (150 mM), cold (4 °C), and PEG6000 (10% w/v). Leaf samples were gathered at specified time points, with 0 h samples acting as the control.

For comparing salt treatment-induced phenotypic changes, nine-week-old plants in similar growth stages were irrigated with a 150 mM NaCl solution every five days for 15 days. Each biological replicate comprised at least 20 plants per genotype across three technical replicates.

### Gene cloning and expression analysis

Sweet cherry leaf total RNA (cultivar ‘Tieton’) was isolated using the Plant RNA Isolation kit from Wolact (Wolact, Vicband Life Sciences Company (Hk) Limited). The cDNAs were obtained using the PrimeScript First-strand cDNA Synthesis kit (TaKaRa, Japan). RT-qPCR analysis followed established methods (Mao et al., 2021), utilizing *PavACTIN* as the internal reference gene (Sun et al., 2023). Three independent biological replicates, each with four technical replicates, were conducted. The primers used in this study are listed in **Table S1**.

### Yeast-two-hybrid and split-LUC assays

To explore PavCBLs’ interaction with PavSOS2, their CDSs were amplified with specific primers (*PavCBLs*-AD-F/R and *SOS2*-BD-F/R) and inserted PCR products into pGADT7 and pGBKT7 vectors. These constructs were transformed into Y2H-Gold strain with specified combinations. Following growth on SD-Trp/-Leu (DDO) medium for two days, positive transformants were cultured on SD-Trp/-Leu/-His/-Ade (QDO; ± X-α-gal) medium (Yang et al., 2023a).

For split-LUC assays, gene CDSs were cloned into pRI101-nLUC and pRI101-cLUC vectors, then expressed in *Nicotiana benthamiana* leaves using the *Agrobacterium*-mediated transient expression method (Mei et al., 2023; Yang et al., 2023a; Yang et al., 2023b). The LUC fluorescence signal was detected using a Lumazone Pylon 2048B imaging system (Roper Scientific, MA, USA).

### Subcellular localization

To determine PavCBL4 protein’s subcellular localization in plant and yeast cells, we used different vectors and transformation methods. *PavCBL4*-GFP vector (controlled by the 35S promoter) was introduced into *Agrobacterium tumefaciens* strain GV3101 for expression in tobacco leaves, as previously described. To identify the PavCBL4 localization in yeast cells, *PavCBL4* CDS was cloned into the pDR196-GFP vector and subsequently transformed it into yeast cells via the LiAc/PEG method. Fluorescence images were obtained using a confocal laser scanning microscope (Leica TCS-SP8 SR).

### Genetic transformation in cherry plants

For the ‘Gisela 6’ seedlings transformation, *PavCBL4* CDS was cloned into pCAMBIA2300-GFP to construct the *PavCBL4*-OE vector. *Agrobacterium rhizogenes* C58C1 was used to genetically introduce the *PavCBL4*-OE vector into ‘Gisela 6’ roots (Sun et al., 2022). Positive transgenic lines were confirmed via genomic PCR analysis of root-extracted total DNA. Additionally, *PavCBL4* expression levels in seedling roots were assessed through qRT-PCR.

### Measurements of stress-related physiological parameters

Relative electrolyte leakage (REL) was calculated as follows: REL = (D1 − D0)/(D2 − D0) × 100 % (Hang et al., 2021; Yang et al., 2021a). Malondialdehyde (MDA) content, superoxide dismutase (SOD), peroxidase (POD), and catalase (CAT) enzymatic activities, H_2_O_2_ and O_2_. ^−^ content, and root activity were measured using their respective kits (Suzhou Comin Biotechnology Co., Ltd, China) following manufacturer’s instructions. DAB (3,3’-diaminobenzidine) and NBT (nitro blue tetrazolium) histochemical staining procedures followed established methods in prior studies (Zhou et al., 2019; Yang et al., 2021b). Total chlorophyll content was measured as described by Liang et al. (2017). The Open FluorCam FC 800-O assessed the maximal quantum efficiency of photosystem II (PSII; Fv/Fm). Na^+^ content was determined as described previously (Yang et al., 2021a; Yang et al., 2023b).

### Statistical analysis

Significant differences among means were analyzed using one-way ANOVA and Student’s *t*-test (*p* < 0.05) via IBM SPSS software (version 26; IBM, Chicago, Illinois, United States).

## Results

### Identification, chromosomal location, cloning, and characterization of PavCBL proteins

**Figure 1:**
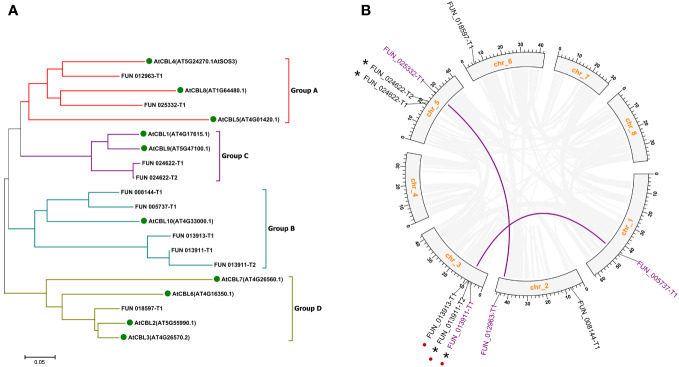
Phylogenic relationships **(A)** and genome locations **(B)** of CBL family genes in sweet cherry. Green dots in **(A)** indicate CBL proteins in *Arabidopsis*. Grayish lines represent collinear blocks. Purple lines indicate the segmental duplication genes. Red dots indicate the tandem duplication genes. Asterisks indicate the different splicing forms of the same gene loci. Chr, chromosome.

To identify PavCBLs in sweet cherry, we employed the HMM file (PF13499.8) as a query to search the *Prunus avium* proteome using the HMMER software, resulting in 150 putative proteins (**Supplementary File 1**). These, along with the 10 AtCBLs (**Supplementary File 2**), were subjected to phylogenetic analysis. The analysis confirmed 10 proteins as sweet cherry CBL family members (**Figure S1**) grouped into four subgroups (A to D), mirroring the CBL family organization in *Arabidopsis* (**Figure 1A**).

Mapping these 10 *PavCBL* genes to the sweet cherry genome (*Prunus avium* Tieton Genome v2.0) revealed their distribution on five of the eight chromosomes (**Figure 1B**). Plant gene family expansion is predominantly due to segmental and tandem duplications (Leister, 2004). Our collinear analysis uncovered intricate patterns of collinearity between chromosomes, identifying two pairs of segmental duplication genes (FUN_012963-T1 and FUN_025332-T1, FUN_013911-T1 and FUN_005737-T1) and one pair of tandem duplication genes (FUN_013911-T1 and FUN_013913-T1) (**Figure 1B**; **Table 1**). FUN_013911-T1/-T2 and FUN_024622-T1/-T2 represent different splicing forms of the gene loci FUN_013911 and FUN_024622 as predicted by the genome (**Table 1**).

**Table 1:** Characterization of the CBL family genes in sweet cherry.

Group	Gene name	Gene locus (GDDH13)	Genomic location (GDDH13)	Deduced polypeptide				Best hits
				Length (aa)	Mass Weight (kDa)	pI	Charge at PH 7.0	
**A**	PavCBL4	FUN_012963-T1 #	chr_2: 44037066 - 44041154	212	24.55	4.65	-14.41	AT5G24270 (CBL4)
	PavCBL8	FUN_025332-T1 #	chr_5: 27504895 - 27507680	240	27.83	5.10	-10.78	AT1G64480 (CBL8)
**B**	×	FUN_008144-T1	chr_2: 7468975 - 7470404	**89**	×	×	×	×
	PavCBL10.1	FUN_005737-T1 #	chr_1: 45784119 - 45786628	246	28.57	4.56	-16.31	AT4G33000 (CBL10)
	PavCBL10.2	FUN_013913-T1	chr_3: 8028538 - 8030748	254	29.27	4.74	-13.27	AT4G33000 (CBL10)
	PavCBL10.3	*FUN_013911-T1 #	chr_3: 8008078 - 8021705	**1081** (266)	(30.60)	(4.77)	(-14.96)	AT4G33000 (CBL10)
	×	*FUN_013911-T2	chr_3: 8008078 - 8021705	**1056**	×	×	×	×
**C**	×	*FUN_024622-T1	chr_5: 22580240 - 22585568	**159**	×	×	×	×
	PavCBL1	*FUN_024622-T2	chr_5: 22580240 - 22585568	213	24.54	4.63	-10.78	AT4G17615 (CBL1)
**D**	PavCBL3	FUN_018597-T1	chr_6: 4425622 - 4429578	226	26.05	4.72	-14.17	AT4G26570 (CBL3)

The protein length, mass weight, isoelectric point (pI) values, and charge at pH 7.0 were determined with the DNAstar software (Editseq, version 7.1.0). The best hits for PavCBL proteins in Arabidopsis were determined by the local BLASTp search with the BioEdit software. The bold format indicates genes with incorrect coding sequences predicted by the sweet cherry genome. Sequence length, mass weight, pI, and Charge at pH7.0 of the revised protein (PavCBL10.3, FUN_013911-T1) are listed in parentheses. The hash marks represent segmental duplication genes, and asterisks indicate different splicing forms of the same gene loci.

Sequence alignments revealed high conservation of CBL protein sequences in sweet cherry and *Arabidopsis*, with a large variation primarily in the N-terminal region (**Figure S2A**). Notably, sequences that were excessively short (FUN_008144-T1, FUN_024622-T1) or long (FUN_013911-T1, FUN_013911-T2) indicated inaccuracies in CDS prediction within the sweet cherry genome, as corroborated by protein sequence alignments (**Figure S2**; **Table 1**). To address these inaccuracies, the coding sequence of FUN_013911-T1 was modified based on the protein sequence alignment (**Figure S2C**). Subsequently, we cloned the remaining 7 out of the 10 *PavCBL* genes from sweet cherry (cultivar ‘Tieton’) and named them according to their *Arabidopsis* orthologs (**Table 1**). The fundamental characteristics of these *PavCBLs* are also provided in **Table 1**.

### Phylogenetic analysis and comparison of gene structure and conserved motif composition patterns of PavCBLs

**Figure 2:**
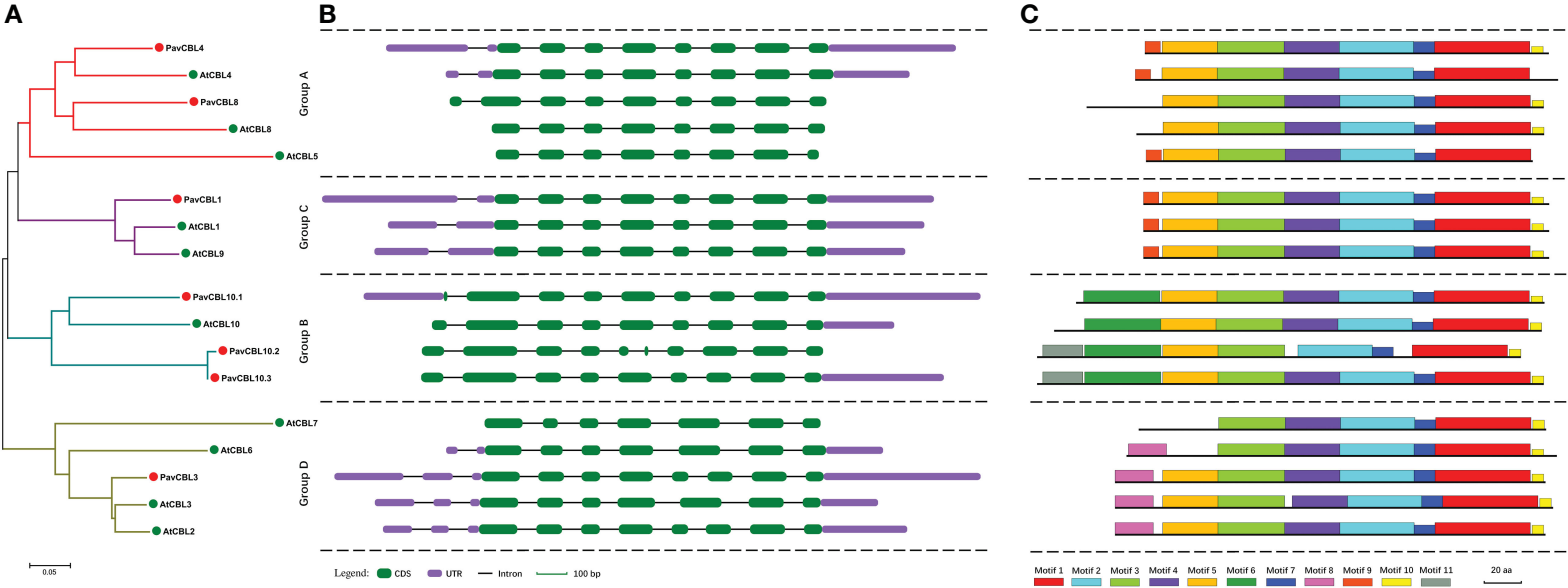
Phylogenic relationships **(A)**, gene structure **(B)**, and conserved motifs **(C)** for the CBL family proteins in sweet cherry and *Arabidopsis*. Green and red dots indicate CBL proteins in *Arabidopsis* (AtCBLs) and sweet cherry (PavCBLs), respectively. Introns are represented by black line segments of the same length to facilitate the comparison of the gene structure composition patterns. Different color boxes represent the 11 conserved motifs.

To enhance comprehension of the correlation amongst PavCBL proteins, we constructed a phylogenetic tree with seven PavCBLs and ten AtCBLs (**Figure 2A**). Results showed that PavCBLs formed four clusters, akin to observations in *Arabidopsis* (**Figure 2A**). Analyzing *PavCBL* gene structures indicated significantly diverse intron lengths (**Figure S3**) and noticeable N-terminal variation among distinct groups, yet genes clustered within the same branch shared similar exon-intron patterns (**Figures 2A, B**). Utilizing MEME software, we also predicted conserved motifs in PavCBLs, with motifs 1, 2, 3, and 7 evident across all proteins (**Figures 2C**; **S4**). However, motif 4 was absent in *PavCBL10.2*, where an analysis revealed a missing fragment in its fifth exon (**Figure 2B**). Additionally, motifs 6 and 8 exclusively appeared in groups B and D, respectively, while motif 9 was solely present in groups A and C (**Figures 2A, C**). These outcomes imply the potential utility of these motifs in differentiating CBL proteins among distinct groups.

### Promoter analysis and expression profile of *PavCBL* genes

**Figure 3:**
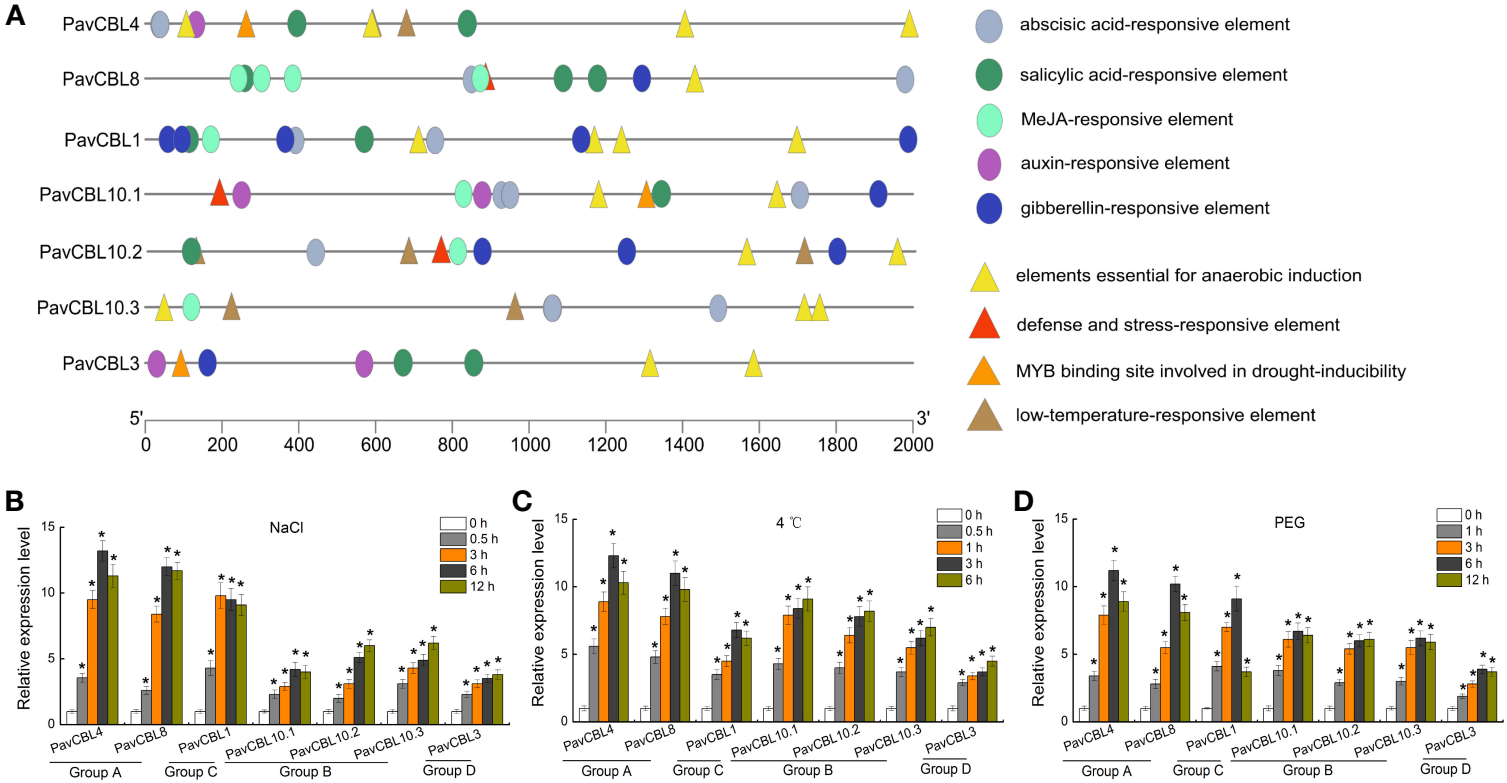
Promoter analysis and expression profile of *PavCBL* genes. **(A)** *Cis*-element analysis of the *PavCBL* genes promoter regions. Circles and triangles indicate the *cis*-elements related to hormone response and abiotic stress response, respectively. **(B–D)** Expression patterns of the *PavCBL* genes in leaves of ‘Gisela 6’ plants under NaCl **(B)**, cold **(C)**, and PEG **(D)** treatments. The expression level was calculated with respect to control samples (0 h) with the 2^—△△CT^ method. * in each panel indicates signifcant differences from the control at P < 0.05.

We obtained the promoter sequences of *PavCBL* genes from the cherry genome and identified the cis-acting elements contained in them using PlantCARE software. These elements, including those linked to hypoxia, low temperature, water deficit, and plant hormones (SA, ABA, MeJA, GA, and auxin). As expected, we discovered numerous abiotic stress and hormone responsive cis-elements (**Figure 3A**).

To delve deeper into *PavCBLs*’ response to abiotic stress, ‘Gisela 6’ seedlings were subjected to hydroponic treatments with NaCl, 4 °C, and PEG6000. qRT-PCR analysis revealed that all *PavCBL* genes were significantly induced by these treatments (**Figures 3B–D**). Additionally, genes classified into the same group exhibited similar expression patterns. Among the CBL family, *PavCBL4* and *PavCBL8* from group A showed the most noticeable changes in expression (**Figures 3B–D**).

### Protein interaction between PavCBLs and PavSOS2

**Figure 4:**
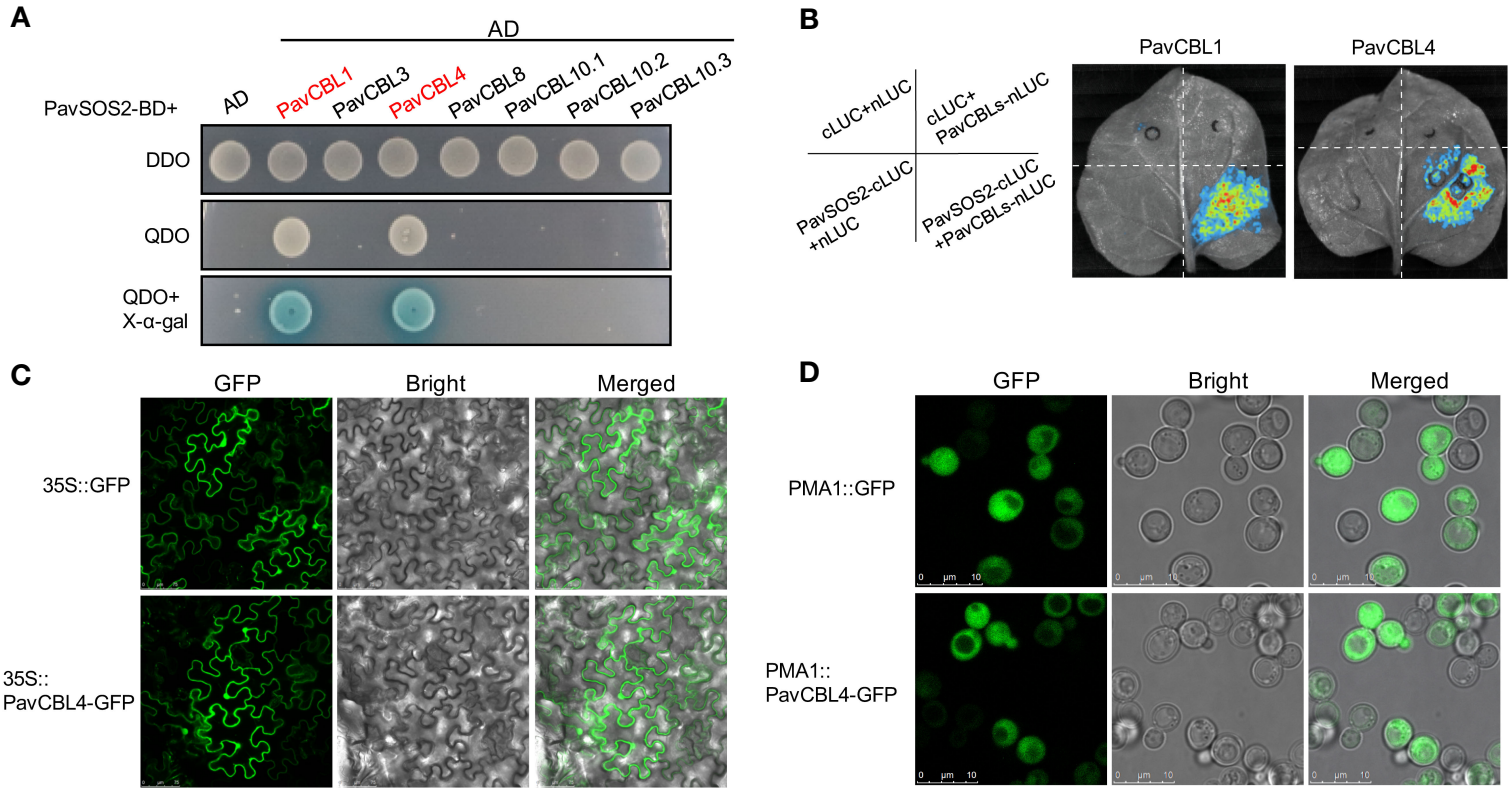
Protein interaction identification between PavCBLs and PavSOS2 and subcellular localization of PavCBL4. **(A)** Identification of the protein interactions between PavCBLs and PavSOS2 through yeast-two-hybrid (Y2H) assays. AD, pGADT7; BD, pGBKT7. DDO, SD medium without leucine and tryptophan; QDO, SD medium without leucine, tryptophan, histidine, and adenine. **(B)** Identification of the protein interactions between PavCBL1/PavCBL4 and PavSOS2 through the Split-LUC assays. **(C)** Subcellular localization of PavCBL4 in epidermal cells of tobacco leaves. Scale bars, 75 μm. **(D)** Subcellular localization of PavCBL4 in yeast cells. Scale bars, 10 μm.

The interaction between CBLs and CIPKs is crucial for plant abiotic stress response. Among these signaling pathways, the SOS pathway has been extensively studied and reportedly positively modulates *Arabidopsis* salt tolerance (Zhu et al., 1998). The SOS2-SOS3/CBL4 complex is a key component of this pathway (Shi et al., 2000; Qiu et al., 2002). To identify the interaction between PavCBLs and PavSOS2, their CDSs were cloned into pGAD-T7 (AD) and pGBK-T7 (BD) vectors, respectively. Y2H assays in yeast indicated that PavCBL1 and PavCBL4 could interact with PavSOS2, while other PavCBLs could not (**Figure 4A**).

To identify protein interactions within a living organism, we conducted Split-LUC assays. The CDSs of *PavSOS2* and *PavCBL1*/4 were cloned into c-LUC and n-LUC vectors, respectively. These constructs were then transiently expressed in tobacco leaves in specified combinations (**Figure 4B**). No fluorescence was detected when PavSOS2 or PavCBL proteins were expressed independently. However, a strong fluorescence signal emerged upon co-expression of PavSOS2 with PavCBL1 (or PavCBL4) (**Figure 4B**), indicating *in vivo* interaction between PavSOS2 and PavCBL1 or PavCBL4.

### Subcellular localization of PavCBL4

Based on expression analysis and protein interaction identification results, *PavCBL4* was selected for further investigation. Certain CBL proteins in Arabidopsis contain a conserved ‘MGCXXSK/T’ motif at their N-terminal, where myristoylation and S-acylation (commonly known as palmitoylation) occur at Gly and Cys residues, respectively. This lipid modification is crucial for membrane localization and function of these CBL proteins (Ishitani et al., 2000; Batistič et al., 2008; Sanyal et al., 2015; Steinhorst et al., 2022). Similar characteristics are observed in other plant CBL proteins like the MdCBL1 in apple (Jiang et al., 2021). Sequence analysis of PavCBL4 showed that it also contained the conserved N-terminal ‘MGCXXSK/T’ motif (**Figure S2A**), suggesting its potential as a membrane-localized protein. To determine PavCBL4’s location in plant cells, PavCBL4-GFP was expressed in tobacco leaves. Surprisingly, the PavCBL4-GFP fluorescence signal was found ubiquitously throughout the cell, indicating localization not only on the membrane but also in the cytoplasm and nucleus (**Figure 4C**). Further validation in yeast cells confirmed the widespread distribution of the green fluorescence signal, excluding vacuoles. Previous studies in Arabidopsis have also shown that AtCBL4 is localized to cytoplasm, nucleus, and plasma membrane, excluding the vacuolar membrane (Batistič et al., 2010). Based on these findings, PavCBL4 appears to be widely distributed in the plant cytoplasm, nucleus, and plasma membrane. Numerous studies have also revealed similar findings. For instance, various CBL proteins with the conserved ‘MGCXXSK/T’ motif are not exclusively localized to the membrane, as observed in AtCBL4 and AtCBL5 in Arabidopsis (Batistič et al., 2010; Sanyal et al., 2015) and MdCBL5 in apple (Gu et al., 2015). The diverse subcellular localization patterns of CBL proteins indicate the complex and varied functions of the CBL family in plant stress response (Batistič et al., 2010; Sanyal et al., 2015).

### *PavCBL4* overexpression in the ‘Gisela 6’ plant roots enhanced salt tolerance

**Figure 5:**
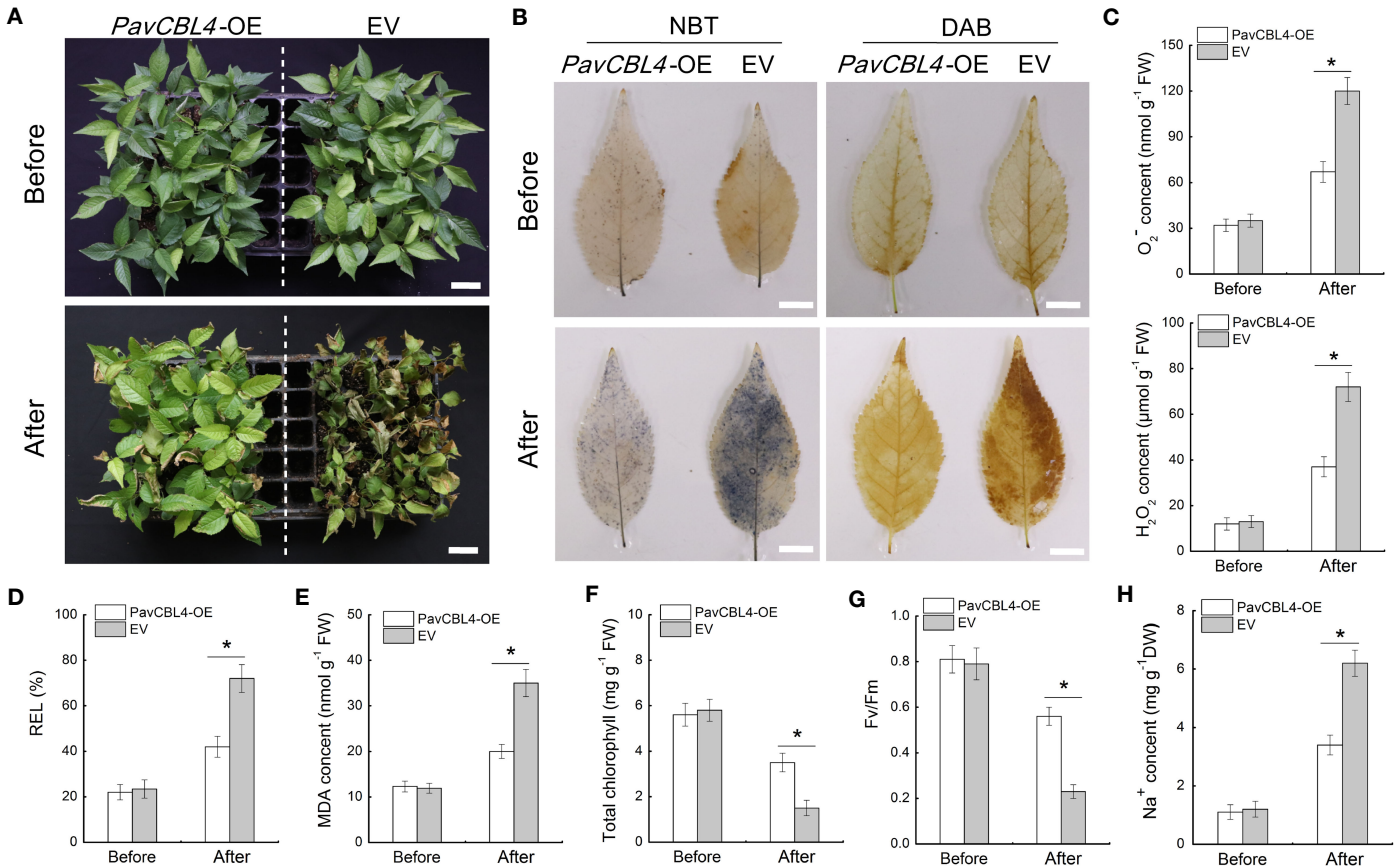
Phenotypic analysis of *PavCBL4*-overexpressing transgenic ‘Gisela 6’ plants under NaCl treatment. **(A)** Growth phenotypes of ‘Gisela 6’ plants before and after NaCl treatment. **(B)** *In situ* accumulation of H_2_O_2_ and O_2_. ^-^ detected by DAB and NBT histochemical staining, respectively. **(C)** H_2_O_2_ and O_2_. ^-^ contents in leaves of ‘Gisela 6’ plants. **(D-H)** Relative electrolyte leakage (REL) **(D)**, malondialdehyde (MDA) content **(E)**, total chlorophyll content **(F)**, Fv/Fm ratio **(G)**, and Na^+^ content **(H)** in leaves of ‘Gisela 6’ plants. * in each panel indicates signifcant differences from the control at P < 0.05.

Utilizing *A. rhizogenes* to create transgenic roots (referred to as ‘composite plants’) provides a convenient method for investigating *PavCBL4* function in cherry plants. Through genomic PCR detection and qRT-PCR analysis, at least 20 composite plants overexpressing *PavCBL4* (*PavCBL4*-OE) in roots were chosen for treatment. Plants transformed with the empty vector (EV) were taken as controls. Initially, no significant genotype-based differences were observed prior to NaCl treatment. After a 15-day treatment period, the *PavCBL4*-OE lines demonstrated superior growth compared to the EV lines (**Figure 5A**). Histological staining employing DAB and NBT revealed lower H_2_O_2_ and O^2−^ levels in the leaves of *PavCBL4*-OE plants compared to EV lines (**Figure 5B**). Quantitative measurements of H_2_O_2_ and O_2_. ^−^ contents corroborated the staining outcomes (**Figure 5C**). These results imply that the OE plants accumulated fewer ROS, thus experiencing reduced oxidative stress compared to EV lines.

For further assessment of salt stress-induced damage, various physiological indices related to stress response were evaluated in the leaves of the plants, including relative electrolyte leakage (REL), MDA content, total chlorophyll content, and Fv/Fm (Yang et al., 2021b). No significant differences in these indices were observed between *PavCBL4*-OE and EV lines prior to salt treatment. Following salt treatment, REL and MDA levels in the *PavCBL4*-OE leaves markedly decreased compared to the EV lines. Additionally, total chlorophyll levels and Fv/Fm significantly increased (**Figures 5D–G**). The results showed that *PavCBL4* overexpression in roots enhanced the salt stress resistance of ‘Gisela 6’ seedlings. Na^+^ content determination results revealed a noticeable decrease in Na^+^ accumulation in *PavCBL4*-OE leaves compared to the EV lines after salt treatment (**Figure 5H**). This suggests that *PavCBL4* overexpression in roots reduced the transport of Na^+^ to the aerial parts of the cherry plants.

### *PavCBL4* overexpression inhibits excessive accumulation of Na^+^ and ROS in roots

**Figure 6:**
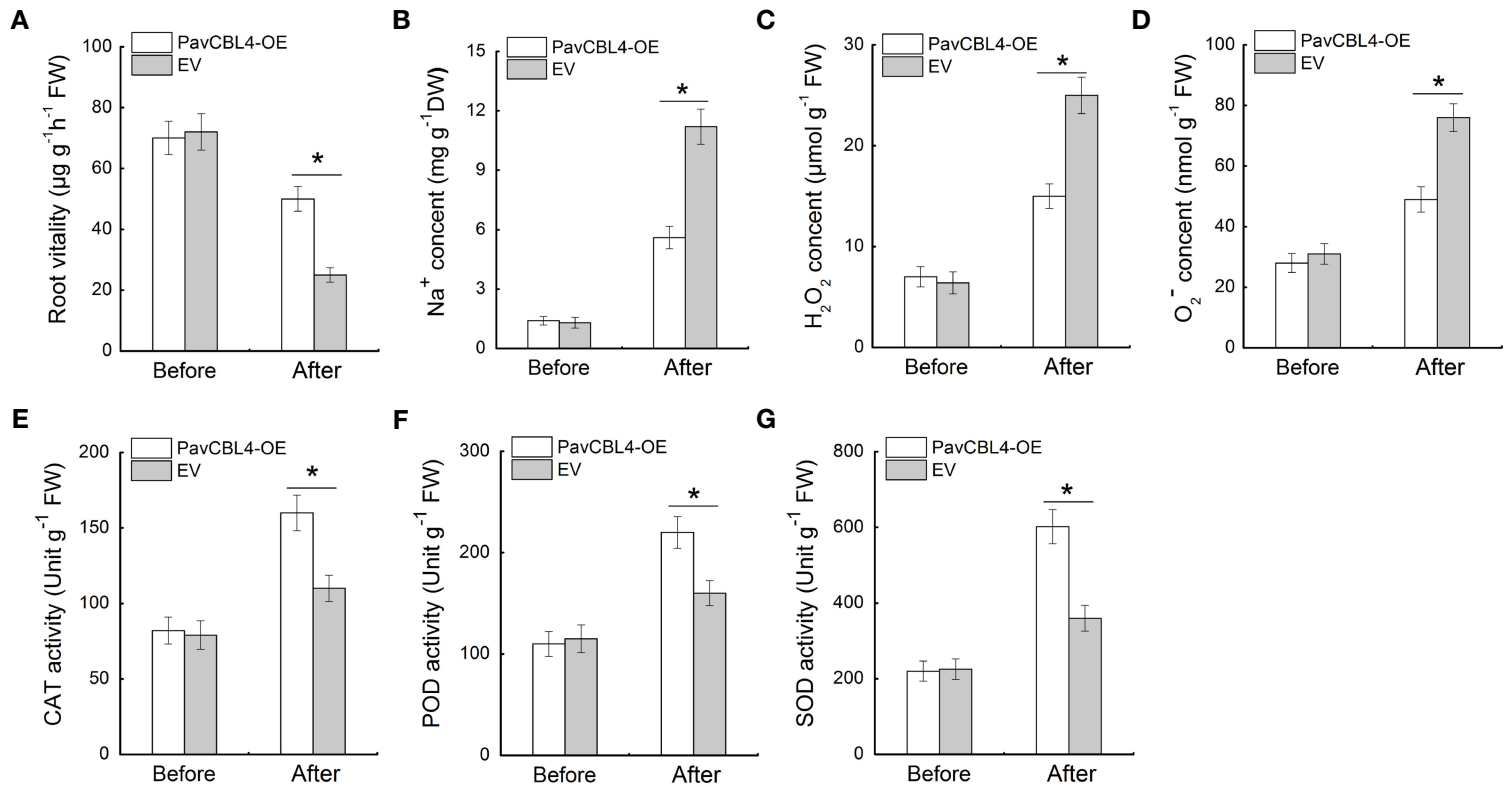
*PavCBL4* overexpression inhibits Na^+^ and ROS accumulation and promotes antioxidant enzyme activities in roots. **(A)** Root vitality of ‘Gisela 6’ plants. **(B)** Na^+^ content in roots. **(C, D)** H_2_O_2_ and O_2_. ^-^ contents in roots of ‘Gisela 6’ plants. **(E–G)** Enzymatic activity of CAT, POD, and SOD antioxidant enzymes. * in each panel indicates signifcant differences from the control at P < 0.05.

Since plant roots are the primary part affected by high salinity, the root vitality was measured. No noticeable difference was observed between the root vitalities of *PavCBL4*-OE and EV lines prior to salt treatment. Post salt stress treatment, *PavCBL4*-OE plants displayed notably higher root vitality compared to EV lines (**Figure 6A**), indicating *PavCBL4* overexpression mitigated salt-induced damage in roots. Additionally, the *PavCBL4*-OE lines exhibited reduced Na^+^ content in roots compared to EV lines under NaCl treatment (**Figure 6B**), demonstrating that *PavCBL4* overexpression suppressed root Na^+^ accumulation.

To investigate the ROS accumulation inhibitory function of *PavCBL4* in cherry plants, the H_2_O_2_ and O_2_. ^−^ levels and antioxidant enzymes’ activity in plant roots were measured. Under salt stress, *PavCBL4*-OE plant roots accumulated lower H_2_O_2_ and O_2_. ^−^ levels compared to EV plants (**Figures 6C, D**). Additionally, OE lines’ roots showed significantly increased antioxidant enzyme activity (**Figures 6E–G**). These results indicated that *PavCBL4* play a positive role in promoting antioxidant enzyme activity and ROS scavenging, thus mitigating oxidative stress damage caused by salt stress.

## Discussion

Plants, due to their stationary growth nature, encounter diverse environmental stressors. Plants have evolved elaborate mechanisms to perceive surrounding environmental changes, orchestrating growth and stress adaptability (Tang et al., 2012). Calcium serves as a crucial nutrient and functions as a second messenger in responding to diverse environmental stresses like water scarcity, extreme temperatures, and high salinity (Yang and Poovaiah, 2003; Kleist and Luan, 2016; Dong et al., 2022). Calcium sensors like Calcineurin B-like (CBL) proteins are essential in plant adaptation to unfavorable environments (Luan, 2009; Dong et al., 2021). However, most studies of CBLs have focused on model plants, and the information on CBLs in woody plants is limited (Kolukisaoglu et al., 2004). Despite being a globally popular fruit, sweet cherry cultivation faces limitations due to various environmental stresses. Therefore, investigating PavCBLs’ regulatory role in responding to salt stress is imperative for the resistance breeding of cherry plants.

Genome-wide screenings in various plant species aimed to uncover CBL proteins crucial in Ca^2+^ signaling and stress response, resulting in diverse CBL member counts. *Arabidopsis*, rice, and *Populus* revealed 10 CBLs each (Zhang et al., 2008; Kleist et al., 2014; Jiang et al., 2020). Cassava, pepper, and pigeon pea identified nine CBLs each (Mo et al., 2018; Ma et al., 2019; Song et al., 2020). Cotton exhibited 13 CBLs (Sun et al., 2021), while tea plant and eggplant showcased 7 CBLs each (Li et al., 2016; Wang et al., 2020b). Beyond stress response, Ca^2+^ is crucial for regulating fruit development, quality, and storage (Picchioni et al., 1998; Chardonnet et al., 2003). The CBL family’s exploration extended to fruit crops like grapevine and pineapple (Xi et al., 2017; Aslam et al., 2019). Nevertheless, a detailed investigation of the CBL family in sweet cherry remains absent. Here, 10 *PavCBL* genes were identified from the sweet cherry genome (**Figures 1**, **S1**; **Table 1**). Collinear analysis unveiled two pairs of segmental duplication genes and one tandem duplication pair (**Figure 1B**; **Table 1**), signifying the contribution of both tandem and segmental duplication events to the CBL family’s expansion during the evolution of sweet cherry.

The polygenetic analysis categorized PavCBL proteins into four groups (**Figures 1A**, **2A**), consistent with grouping patterns observed in CBLs of *Arabidopsis* (Kleist et al., 2014) and various other plant species (Zhang et al., 2008; Jiang et al., 2020; Chen et al., 2021). The phylogenetic tree (**Figure 2A**) was corroborated by comparing gene structure compositions and conserved motifs of AtCBLs and PavCBLs across distinct groups (**Figures 2B, C**). In addition, several unique characteristics, such as differing intron counts in the N-terminal region and exclusive conserved motifs in specific groups (**Figures 2B, C**; **Figure S4**), were identified among these CBL family members. Similar results have been observed in studies on CBL proteins, exemplified by MdCBLs in apple (Chen et al., 2021). Therefore, these discoveries establish a foundation for discerning CBL proteins within groups and exploring interrelationships among these CBLs in plants.

Environmental stress significantly induced the expression of various *CBL* genes (Kudla et al., 1999; Aslam et al., 2019; Dong et al., 2021). Stress reportedly induces a transient elevation of intracellular Ca^2+^ levels, which triggers CBL proteins’ response (Yang and Poovaiah, 2003; Dong et al., 2022). Ca^2+^ binding by CBLs facilitate their interactions CIPKs (Cheong et al., 2007; Li et al., 2016), notably activating the SOS pathway under salt stress (Qiu et al., 2002; Luan, 2009; Wang et al., 2020b; Yan et al., 2021). In *Arabidopsis*, the SOS pathway include SOS3 (CBL4) from the CBL family, SOS2 (CIPK24) from the CIPK family, and SOS1 (NHX7) from the NHX (Na^+^/H^+^ antiporter) family (Qiu et al., 2002; Sanchez-Barrena et al., 2005). High salinity activates the SOS pathway, which promotes Na^+^ efflux, thereby mitigating Na^+^ over-accumulation-induced damage (Qiu et al., 2002; El-Dakak et al., 2021). The positive role of SOS2 in salt stress response and CBLs-SOS2 interactions have been identified in various crops like pepper (Ma et al., 2023), poplar (Tang et al., 2014), and apple (Hu et al., 2012; Hu et al., 2016). This study detected several stress and hormone response-related cis-elements in *PavCBLs* promoters (**Figure 3A**). Furthermore, *PavCBLs*’ transcription levels were obviously up-regulated in response to abiotic stress treatments, especially *PavCBL4* and *PavCBL8* in group A (**Figure 3B**). The outcomes, along with the PavCBL4-PavSOS2 interaction (**Figure 4**), suggested that *PavCBL4* may positively regulate sweet cherry salt response by curtailing excessive Na^+^ accumulation through the SOS pathway. The role of *PavCBL4* in modulating sweet cherry salt tolerance was thus investigated using transgenic ‘Gisela 6’ plants, and the Na^+^ content in roots of was measured. Our findings demonstrate a significant enhancement in salt tolerance in transgenic plants, accompanied by a significant reduction in root Na^+^ accumulation (**Figure 6B**), suggesting a positive role of *PavCBL4* in salt stress response. The lower Na^+^ content in leaves may be attributed to decreased root Na^+^ absorption, leading to lower Na^+^ transport to the aerial parts (**Figure 5H**).

Besides the NHX transporters within the SOS pathway, CBL-activated CIPKs interact with and phosphorylate downstream substrates, including ion transporters and enzymes (Xu et al., 2006; Yang et al., 2019; Yan et al., 2021; Ju et al., 2022). This coordination encompasses various plant stress responses, including Na^+^/K^+^ balance, osmoregulatory substance accumulation, and antioxidant enzyme activity (Xu et al., 2006; Liu et al., 2013; Ju et al., 2022). Numerous studies indicated that overexpression of *SOS2* or *CBLs* in plants under abiotic stress conditions increases antioxidant enzyme activity, e.g., *PtSOS2* in poplar (Zhou et al., 2014), and *MdSOS2L1* (Hu et al., 2016) and *MdCBL10.1* (Chen et al., 2021) in apple. In *PavCBL4*-OE transgenic roots, antioxidant enzyme activity was higher than those of controls under salt stress (**Figures 6E–G**). Furthermore, *PavCBL4*-OE transgenic roots exhibited significantly lower ROS levels than control roots (**Figures 6C, D**). These results suggested that, in addition to preventing excessive Na^+^ accumulation, *PavCBL4* enhances cherry plant salt tolerance by improving antioxidant enzyme activity. Overall, this study provides valuable insights for future investigations into the mechanisms underlying *PavCBLs*-mediated salt stress responses in sweet cherry.

## Data availability statement

The original contributions presented in the study are included in the article/**Supplementary Material**. Further inquiries can be directed to the corresponding authors.

## Author contributions

QF: Writing – original draft. SH: Writing – review & editing. RG: Writing – review & editing. GW: Writing – review & editing. YS: Writing – review & editing.

## Supplementary material

File 2 Protein sequences of the HMMER screening results and the 10 AtCBLs.
