# Supplementary material for: Functional identification of the calcineurin B-like protein PavCBL4 in modulating salt tolerance in sweet cherry
Source: Front Plant Sci. 2023 Nov 22;14:1293167. doi: 10.3389/fpls.2023.1293167 (PMC10702776; doi:10.3389/fpls.2023.1293167)
Supplement: File 2 — Protein sequences of the HMMER screening results and the 10 AtCBLs. [file DataSheet_1.pdf]

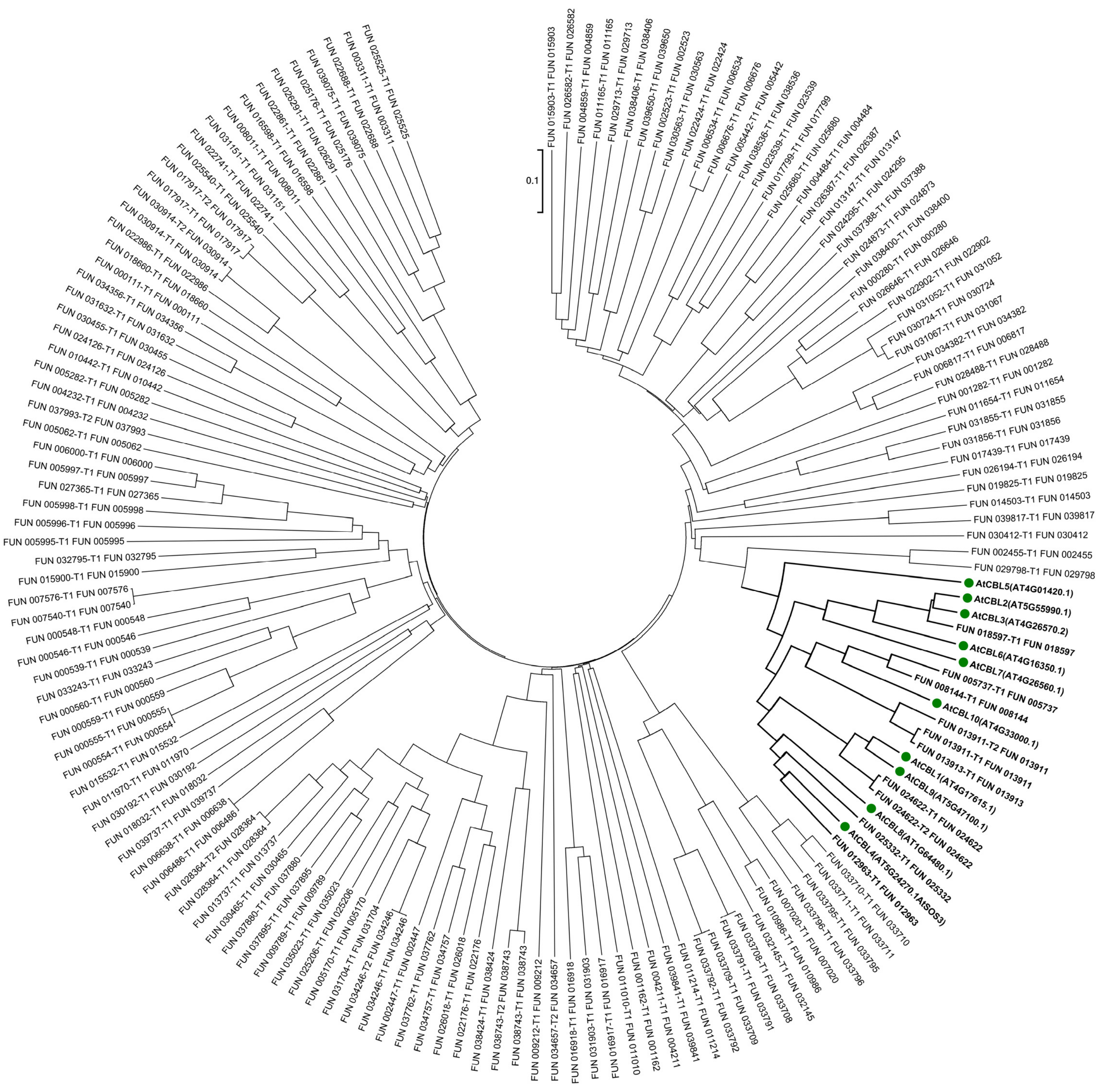

Figure S1 Phylogenic analysis of AtCBLs in *Arabidopsis* and proteins in HMMER screening results.

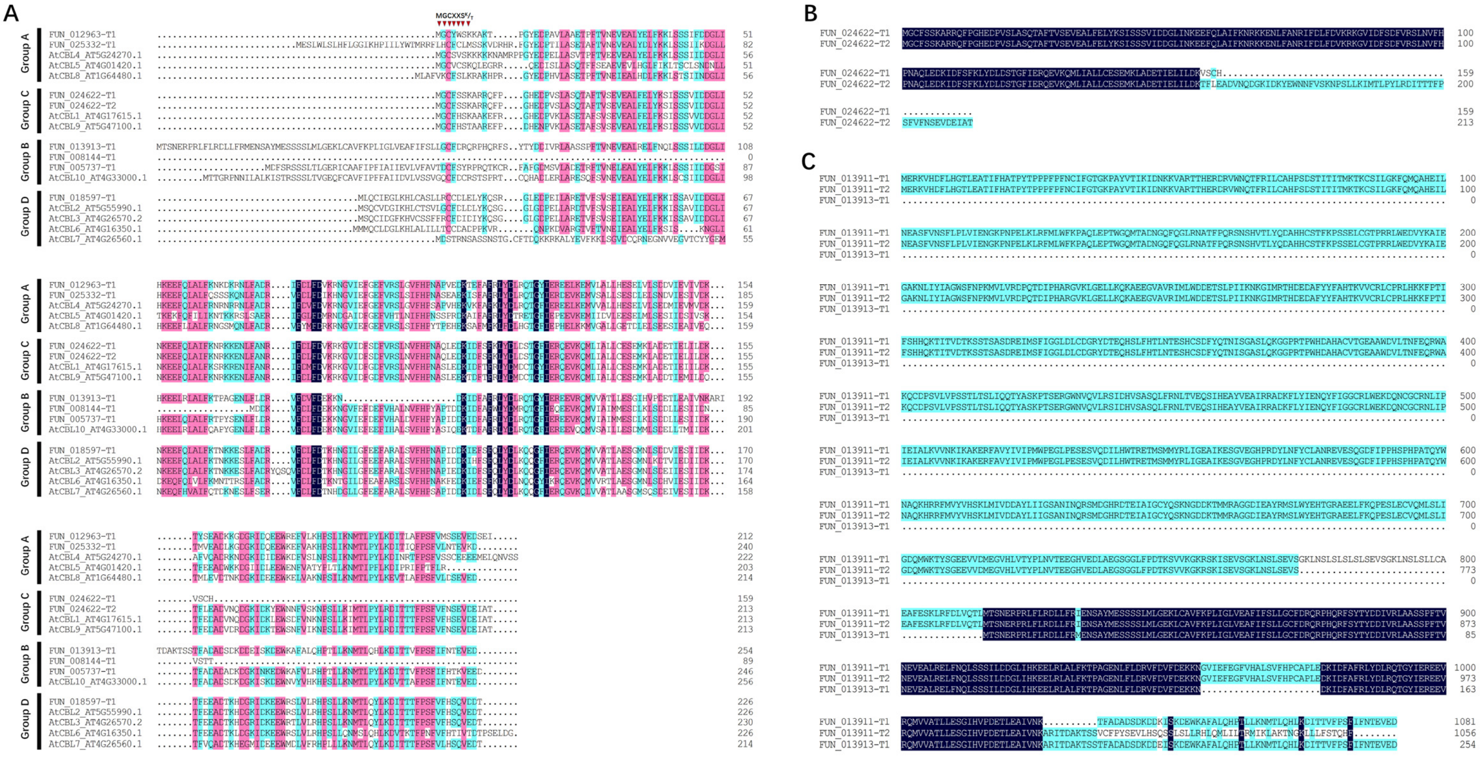

Figure S2 Sequence alignments of AtCBLs and putative PaCBLs identified in the sweet cherry genome.

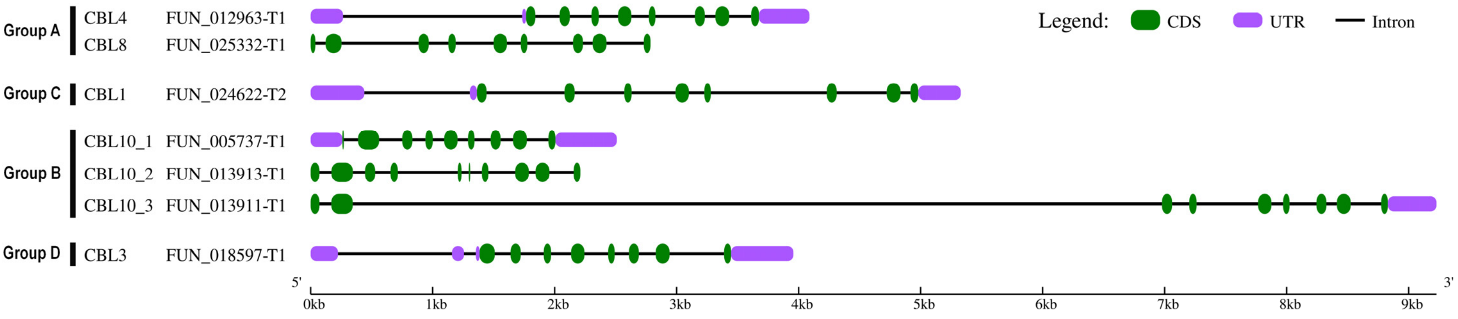

Figure S3 Gene structure of CBL family genes in sweet cherry.

Motif	Logo	E-value	Sites	Width
1	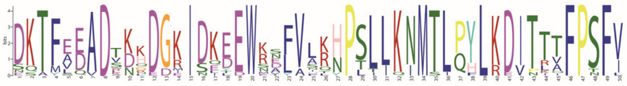	9.7e-475	17	50
2	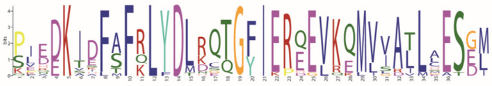	1.2e-337	17	39
3	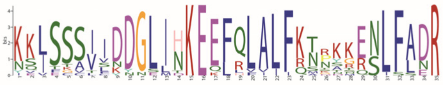	2.1e-257	17	35
4	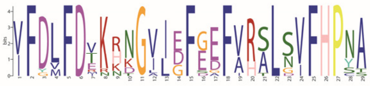	1.1e-245	16	29
5	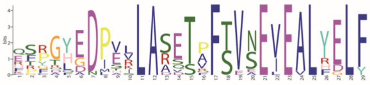	3.8e-165	15	29
6	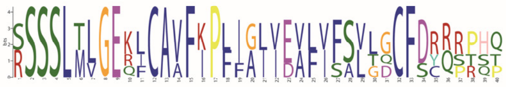	2.7e-026	4	40
7	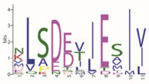	3.5e-025	17	11
8	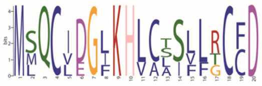	3.3e-020	4	20
9	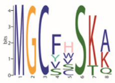	1.7e-012	6	8
10	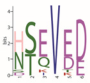	2.4e-008	15	6
11	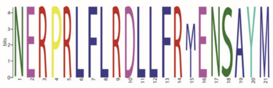	8.4e-003	2	21

Figure S4 Conserved motifs in the PavCBL proteins identified via the online MEME software.

Table S1 The primers used in this study.

Annotation	Primer name	Sequence(5'-3')
Y2H analysis	PavSOS2-BD-F	CGGAATTCCCGGGGATCC TGAAGAAGGTAGGGAAGT
	PavSOS2-BD-R	GCTTGGCTGCAGGTCGAC TCAGCAAGTCATTGTTCG
	PavCBL4-AD-F	TCGAATTCCCGGGGATCC TGGGCTGCTATTGGTCAA
	PavCBL4-AD-R	AGATCTCTGCAGGTCGAC TCATATTTCTGAATCTTC
	PavCBL8-AD-F	TCGAATTCCCGGGGATCC TGGAAAGTTTGTGGCTCA
	PavCBL8-AD-R	AGATCTCTGCAGGTCGAC CTAATCTTTAACTTCAGT
	PavCBL1-AD-F	TCGAATTCCCGGGGATCC TGGGTTGTTTTAGTTCTA
	PavCBL1-AD-R	AGATCTCTGCAGGTCGAC TCACGTAGCAATCTCATC
	PavCBL10.1-AD-F	TCGAATTCCCGGGGATCC TGGATTTCTCGAGATCGA
	PavCBL10.1-AD-R	AGATCTCTGCAGGTCGAC TCAGTCTTCTTCAACTTT
	PavCBL10.2-AD-F	TCGAATTCCCGGGGATCC TGACGAGCAATGAGAGAC
	PavCBL10.2-AD-R	AGATCTCTGCAGGTCGAC TTAGTCTTCGACTTCGGT
	PavCBL10.3-AD-F	TCGAATTCCCGGGGATCC TGACGAGCAATGAGAGAC
	PavCBL10.3-AD-R	AGATCTCTGCAGGTCGAC TTAGTCTTCGACTTCGGT
	PavCBL3-AD-F	TCGAATTCCCGGGGATCC TGTTGCAGTGCATAGAGG
	PavCBL3-AD-R	AGATCTCTGCAGGTCGAC TCAGGTGTCGTCCACTTG
Split-LUC analysis	PavSOS2-cLUC-F	AAAGCAGGCTTCGGATCC ATGAAGAAGGTAGGGAAGT
	PavSOS2-cLUC-R	GTTGTTGATTCAGAATTC TCAGCAAGTCATTGTTCG
	PavCBL1-nLUC-F	ATACATATGCCCGTCGAC ATGGGTTGTTTTAGTTCT
	PavCBL1-nLUC-R	GAAAGCTGGGTTGGTACC CGTAGCAATCTCATCAAC
	PavCBL4-nLUC-F	ATACATATGCCCGTCGAC ATGGGCTGCTATTGGTCA
	PavCBL4-nLUC-R	GAAAGCTGGGTTGGTACC TATTTCTGAATCTTCAAC
Genetic transformation	PavCBL4-pCambia2300-GFP-F	AACACGGGGGACGAGCTC ATGGGCTGCTATTGGTCA
	PavCBL4-pCambia2300-GFP-R	CTCTAGAGGATCCCCGGG ATATTTCTGAATCTTCAA
qRT-PCR	PavCBL4--qF	GTCTTTCACCCTAATGCACCTGTAG
	PavCBL4--qR	TCCTTCAACTCCTCTCGCTCAATG
	PavCBL8--qF	TACATTGAGCGTGATGAGGTGAAGG
	PavCBL8--qR	TCTGCCTCCACCATTGTCTTATCG
	PavCBL1--qF	TCTTCCATCCCAATGCCCAACTAG
	PavCBL1--qR	CATCAGCCAGCTTCATTTCAGACTC
	PavCBL10.1--qF	CTTTAACATTGGGCGAGCGGATC
	PavCBL10.1--qR	GGACGGTAGCTGAAGCAATCGG
	PavCBL10.2--qF	CGCTTCTCCTACACTTACGACGAC
	PavCBL10.2--qR	GCTCCTCCTTGTGAATTAAGCCATC
	PavCBL10.3--qF	CGCTTCTCCTACACTTACGACGAC
	PavCBL10.3--qR	GCTCCTCCTTGTGAATTAAGCCATC
	PavCBL3--qF	GCCCTCTCTGTCTTTCATCCTAACG
	PavCBL3--qR	CATTTGCTTCACCTCCTGCCTTTC

	PavACTIN--qF	CCAGGGCTGTGTTTCCTTCTA
	PavACTIN--qR	ATGATCTGCGTCATCTTTTCT

The red letters refer to the nucleotide sequences in the vectors.

File1 Overview of the HMMER screening results.

	Full sequence			best 1 domain			dom			
	**E-value**	score	bias	E-value	score	bias	exp	N	**Sequence**	Description
1	**2.60E-36**	125	6.6	1.70E-19	71.1	1.5	2.1	2	**FUN_000280-T1**	FUN_000280
2	**1.20E-34**	119.7	5.5	8.10E-18	65.8	1.8	2.1	2	**FUN_026646-T1**	FUN_026646
3	**1.10E-32**	113.4	0.2	3.90E-15	57.2	0.1	2.2	2	**FUN_024295-T1**	FUN_024295
4	**3.20E-32**	111.9	9.1	2.20E-13	51.6	2.1	3.9	4	**FUN_038406-T1**	FUN_038406
5	**2.30E-31**	109.2	4.4	3.60E-17	63.7	1.1	3	2	**FUN_022902-T1**	FUN_022902
6	**1.50E-30**	106.5	7.8	2.00E-15	58.1	0.5	2.1	2	**FUN_038400-T1**	FUN_038400
7	**1.50E-30**	106.5	10.3	4.70E-14	53.7	2.5	3.2	3	**FUN_005170-T1**	FUN_005170
8	**2.00E-30**	106.2	0.3	3.20E-14	54.3	0.1	2.4	2	**FUN_013147-T1**	FUN_013147
9	**2.70E-30**	105.7	3.7	7.50E-16	59.5	1	2.5	2	**FUN_035023-T1**	FUN_035023
10	**5.80E-30**	104.7	3.6	5.40E-17	63.1	0.8	2.1	2	**FUN_031067-T1**	FUN_031067
11	**1.20E-29**	103.7	3.4	5.40E-17	63.1	0.8	2.1	2	**FUN_030724-T1**	FUN_030724
12	**2.60E-29**	102.6	0	1.00E-15	59.1	0	2.1	2	**FUN_026387-T1**	FUN_026387
13	**9.80E-29**	100.7	12.7	1.10E-13	52.5	0.3	2.8	3	**FUN_029713-T1**	FUN_029713
14	**2.90E-28**	99.2	12.3	8.50E-12	46.5	0.6	2.6	3	**FUN_015903-T1**	FUN_015903
15	**3.20E-28**	99.1	0.1	6.40E-15	56.5	0	2.7	2	**FUN_004484-T1**	FUN_004484
16	**7.40E-28**	97.9	3.3	3.80E-14	54	3.4	2.5	2	**FUN_034246-T1**	FUN_034246
17	**8.40E-28**	97.7	6.1	3.60E-14	54.1	0.7	2.1	2	**FUN_030563-T1**	FUN_030563
18	**1.10E-27**	97.4	10.9	1.10E-13	52.6	2.2	2.3	2	**FUN_039650-T1**	FUN_039650
19	**1.80E-27**	96.7	3	1.40E-13	52.2	0.7	3	3	**FUN_031704-T1**	FUN_031704
20	**2.70E-27**	96.2	6	5.20E-13	50.4	0.9	2.1	2	**FUN_002523-T1**	FUN_002523
21	**2.80E-27**	96.1	3.6	9.80E-14	52.7	1.5	2.7	2	**FUN_025206-T1**	FUN_025206
22	**2.80E-27**	96.1	5.4	7.90E-14	53	2.3	2.5	2	**FUN_022176-T1**	FUN_022176
23	**5.20E-27**	95.2	2.9	1.50E-13	52.1	1.9	2.6	2	**FUN_026018-T1**	FUN_026018
24	**2.10E-26**	93.3	4.5	1.00E-13	52.7	2.5	2.6	2	**FUN_037880-T1**	FUN_037880
25	**2.40E-26**	93.1	1.1	7.80E-12	46.6	0.1	2.1	2	**FUN_004859-T1**	FUN_004859
26	**2.90E-26**	92.9	5.5	1.40E-13	52.2	1.9	2.7	2	**FUN_037895-T1**	FUN_037895
27	**3.00E-26**	92.8	4.3	1.60E-12	48.8	0.6	2.9	2	**FUN_030465-T1**	FUN_030465
28	**3.90E-26**	92.4	6.9	6.70E-12	46.8	0.8	3	3	**FUN_028364-T1**	FUN_028364
29	**9.00E-26**	91.3	0.6	6.70E-12	46.8	0	2.3	2	**FUN_011165-T1**	FUN_011165
30	**1.20E-25**	90.8	1.2	7.20E-17	62.7	0.1	4.7	4	**FUN_030914-T2**	FUN_030914
31	**1.30E-25**	90.8	2.9	4.30E-13	50.6	1.4	2.6	2	**FUN_009789-T1**	FUN_009789
32	**2.30E-25**	90	0.3	5.50E-13	50.3	0.1	2.2	2	**FUN_023539-T1**	FUN_023539
33	**3.00E-25**	89.6	2.4	9.80E-17	62.3	0.1	5.2	4	**FUN_030914-T1**	FUN_030914
34	**1.30E-24**	87.5	0.7	2.40E-12	48.3	1.2	2.8	2	**FUN_034757-T1**	FUN_034757
35	**4.20E-24**	85.9	3.4	3.30E-12	47.8	0.1	2.8	2	**FUN_026582-T1**	FUN_026582
36	**4.50E-24**	85.8	0.3	3.10E-13	51.1	0.1	2.1	2	**FUN_025680-T1**	FUN_025680
37	**2.70E-23**	83.3	8.4	3.80E-18	66.8	1.3	4.6	3	**FUN_022986-T1**	FUN_022986
38	**3.80E-23**	82.8	2.6	1.40E-10	42.6	0.4	2.3	2	**FUN_031052-T1**	FUN_031052
39	**7.10E-23**	82	3.2	4.80E-10	40.9	1.7	2.1	2	**FUN_037388-T1**	FUN_037388
40	**7.90E-23**	81.8	7.6	4.50E-11	44.2	1.9	2.8	2	**FUN_013737-T1**	FUN_013737
41	**2.70E-22**	80.1	6.6	8.20E-11	43.3	3.1	2.5	2	**FUN_033711-T1**	FUN_033711

42	**3.80E-22**	79.7	0.1	5.90E-14	53.4	0.3	2.1	2	**FUN_024873-T1**	FUN_024873
43	**5.50E-22**	79.1	6	1.00E-12	49.4	1.7	2.6	2	**FUN_038536-T1**	FUN_038536
44	**5.70E-22**	79.1	0.6	1.50E-09	39.3	0	2.1	2	**FUN_006817-T1**	FUN_006817
45	**1.20E-21**	78	3.8	5.30E-13	50.3	1.6	2.8	2	**FUN_037762-T1**	FUN_037762
46	**1.30E-21**	77.9	3.4	9.80E-14	52.7	0.1	2.8	3	**FUN_009212-T1**	FUN_009212
47	**1.90E-21**	77.4	3	1.30E-09	39.5	0.2	2.6	2	**FUN_038743-T1**	FUN_038743
48	**2.00E-21**	77.3	7.2	1.70E-10	42.3	3.5	2.5	2	**FUN_033710-T1**	FUN_033710
49	**3.50E-21**	76.5	7.2	1.60E-09	39.2	3.4	2.5	2	**FUN_033796-T1**	FUN_033796
50	**2.10E-20**	74	6.3	5.40E-13	50.3	2.2	2.4	2	**FUN_005442-T1**	FUN_005442
51	**2.50E-20**	73.8	8.3	8.50E-10	40.1	0	3.8	4	**FUN_005062-T1**	FUN_005062
52	**1.10E-19**	71.7	5.3	9.60E-10	39.9	1.8	2.4	2	**FUN_000111-T1**	FUN_000111
53	**7.80E-19**	69	10.7	9.50E-11	43.1	3.5	4	4	**FUN_002447-T1**	FUN_002447
54	**3.90E-18**	66.8	2.5	1.30E-12	49.1	0.3	1.7	2	**FUN_015900-T1**	FUN_015900
55	**9.30E-18**	65.6	8.2	1.30E-11	45.9	1.1	2.4	2	**FUN_022424-T1**	FUN_022424
56	**1.90E-17**	64.6	7.3	7.60E-05	24.2	0	4.9	4	**FUN_006638-T1**	FUN_006638
57	**1.90E-17**	64.6	7.3	7.60E-05	24.2	0	4.9	4	**FUN_006486-T1**	FUN_006486
58	**2.70E-17**	64.1	8	6.70E-08	34	0.5	2.5	2	**FUN_018660-T1**	FUN_018660
59	**3.20E-17**	63.9	2.4	3.40E-09	38.2	0	3.1	2	**FUN_017799-T1**	FUN_017799
60	**3.20E-17**	63.9	2.4	8.40E-10	40.1	0.3	2.4	2	**FUN_017917-T2**	FUN_017917
61	**4.10E-17**	63.5	2.4	9.80E-10	39.9	0.3	2.4	2	**FUN_017917-T1**	FUN_017917
62	**5.40E-17**	63.1	1.4	5.20E-10	40.8	0.4	2.2	2	**FUN_001282-T1**	FUN_001282
63	**2.40E-16**	61.1	9.2	1.10E-10	42.9	0.9	2.9	2	**FUN_006676-T1**	FUN_006676
64	**4.30E-16**	60.2	7.4	0.0037	18.8	0	5	4	**FUN_039737-T1**	FUN_039737
65	**4.70E-16**	60.1	8.7	1.20E-10	42.8	0.9	2.9	2	**FUN_006534-T1**	FUN_006534
66	**2.70E-15**	57.7	2.8	5.40E-10	40.7	0.1	2.5	2	**FUN_014503-T1**	FUN_014503
67	**4.00E-15**	57.1	0	5.00E-06	28	0	3.1	3	**FUN_030412-T1**	FUN_030412
68	**6.10E-15**	56.6	9.2	2.10E-08	35.6	1.2	3.6	4	**FUN_018597-T1**	FUN_018597
69	**2.20E-14**	54.8	1.6	2.80E-08	35.2	0.4	2.4	2	**FUN_025540-T1**	FUN_025540
70	**9.10E-14**	52.8	3	9.10E-14	52.8	3	2	1	**FUN_017439-T1**	FUN_017439
71	**3.40E-13**	51	4.2	3.70E-08	34.8	0.2	2.5	2	**FUN_039817-T1**	FUN_039817
72	**3.60E-13**	50.9	13.1	7.20E-08	33.9	3.8	2.9	2	**FUN_005737-T1**	FUN_005737
73	**5.90E-13**	50.2	6	1.50E-08	36.1	0.5	2.7	2	**FUN_025332-T1**	FUN_025332
74	**1.50E-12**	48.9	0.3	2.80E-10	41.6	0	2.6	2	**FUN_034246-T2**	FUN_034246
75	**1.50E-12**	48.9	10.9	1.20E-08	36.4	1.4	3.1	3	**FUN_012963-T1**	FUN_012963
76	**2.20E-12**	48.4	0.4	2.70E-12	48.1	0.4	1.1	1	**FUN_032795-T1**	FUN_032795
77	**7.70E-12**	46.6	7.8	9.40E-07	30.3	2.3	2.7	2	**FUN_024622-T2**	FUN_024622
78	**8.90E-12**	46.4	0.2	1.50E-11	45.7	0.2	1.3	1	**FUN_016598-T1**	FUN_016598
79	**1.20E-11**	46	1.4	2.60E-11	44.9	0.3	1.9	1	**FUN_026194-T1**	FUN_026194
80	**1.90E-11**	45.3	2.4	3.70E-11	44.4	1.9	1.8	1	**FUN_011010-T1**	FUN_011010
81	**2.80E-11**	44.8	0.7	5.00E-11	44	0.5	1.5	1	**FUN_008011-T1**	FUN_008011
82	**3.40E-11**	44.5	5.3	8.50E-08	33.7	0.6	2.8	2	**FUN_013911-T1**	FUN_013911
83	**3.50E-11**	44.5	3.5	9.80E-11	43.1	3.5	1.8	1	**FUN_033795-T1**	FUN_033795
84	**1.60E-10**	42.4	6.3	1.10E-06	30.1	1.6	2	2	**FUN_000559-T1**	FUN_000559
85	**3.60E-10**	41.3	0.6	1.00E-09	39.8	0.6	1.8	1	**FUN_001162-T1**	FUN_001162

86	**5.10E-10**	40.8	3.4	1.00E-09	39.8	3.1	1.7	1	**FUN_033791-T1**	FUN_033791
87	**5.10E-10**	40.8	3.4	1.00E-09	39.8	3.1	1.6	1	**FUN_033708-T1**	FUN_033708
88	**7.80E-10**	40.2	0.3	2.00E-06	29.3	0	2.2	2	**FUN_028488-T1**	FUN_028488
89	**8.30E-10**	40.1	0.5	0.00098	20.6	0	2.1	2	**FUN_034382-T1**	FUN_034382
90	**1.20E-09**	39.6	2	1.70E-06	29.5	0	3.8	4	**FUN_030192-T1**	FUN_030192
91	**1.80E-09**	39	6.5	0.0087	17.6	0.1	3.2	3	**FUN_005995-T1**	FUN_005995
92	**2.20E-09**	38.8	0.3	0.0032	19	0	2.6	2	**FUN_019825-T1**	FUN_019825
93	**3.50E-09**	38.1	7.2	1.00E-05	27	0.6	2	2	**FUN_008144-T1**	FUN_008144
94	**4.70E-09**	37.7	3.2	1.10E-08	36.5	2.3	2	1	**FUN_004211-T1**	FUN_004211
95	**5.80E-09**	37.4	2.2	1.10E-08	36.4	2.1	1.6	1	**FUN_033709-T1**	FUN_033709
96	**5.80E-09**	37.4	2.2	1.10E-08	36.4	2.1	1.6	1	**FUN_033792-T1**	FUN_033792
97	**9.90E-09**	36.7	6.9	0.00099	20.6	0.5	2.5	3	**FUN_024622-T1**	FUN_024622
98	**1.30E-08**	36.3	8	4.40E-06	28.2	2.4	2.2	2	**FUN_000554-T1**	FUN_000554
99	**1.30E-08**	36.3	8	4.40E-06	28.2	2.4	2.2	2	**FUN_000555-T1**	FUN_000555
100	**1.30E-08**	36.3	0.2	6.20E-08	34.1	0.2	1.9	1	**FUN_005996-T1**	FUN_005996
101	**2.40E-08**	35.4	1.1	1.70E-06	29.5	0.1	3	2	**FUN_007540-T1**	FUN_007540
102	**2.40E-08**	35.4	1.1	1.70E-06	29.5	0.1	3	2	**FUN_007576-T1**	FUN_007576
103	**4.10E-08**	34.7	0.7	0.0021	19.6	0	2.8	2	**FUN_013911-T2**	FUN_013911
104	**4.40E-08**	34.6	0.2	6.80E-08	34	0.2	1.3	1	**FUN_010986-T1**	FUN_010986
105	**5.00E-08**	34.4	1.6	8.70E-08	33.6	1.6	1.4	1	**FUN_015532-T1**	FUN_015532
106	**8.20E-08**	33.7	4.6	0.00012	23.6	0.2	3.3	2	**FUN_022688-T1**	FUN_022688
107	**8.40E-08**	33.7	4.9	0.00016	23.2	0.3	2.4	2	**FUN_025525-T1**	FUN_025525
108	**8.90E-08**	33.6	0.7	1.70E-06	29.5	0.1	3.1	1	**FUN_031632-T1**	FUN_031632
109	**9.90E-08**	33.5	0.1	0.0049	18.4	0	2.5	2	**FUN_033243-T1**	FUN_033243
110	**1.00E-07**	33.4	6.2	7.40E-07	30.6	0.7	3.4	2	**FUN_013913-T1**	FUN_013913
111	**1.00E-07**	33.4	1.7	0.00028	22.4	0.2	2.8	2	**FUN_011214-T1**	FUN_011214
112	**1.60E-07**	32.8	0.3	2.70E-06	28.9	0.3	2.2	1	**FUN_006000-T1**	FUN_006000
113	**2.50E-07**	32.2	0.4	0.0011	20.4	0	2.4	2	**FUN_000560-T1**	FUN_000560
114	**3.30E-07**	31.8	1.4	7.40E-06	27.4	0.2	2	2	**FUN_027365-T1**	FUN_027365
115	**5.60E-07**	31	4.9	0.00014	23.4	0.2	2.5	3	**FUN_003311-T1**	FUN_003311
116	**5.80E-07**	31	4.1	0.019	16.5	0.3	3.5	2	**FUN_030455-T1**	FUN_030455
117	**6.40E-07**	30.8	5.1	0.00014	23.4	0.2	2.7	2	**FUN_025176-T1**	FUN_025176
118	**9.10E-07**	30.4	0.3	1.40E-05	26.5	0.3	2	1	**FUN_005997-T1**	FUN_005997
119	**1.60E-06**	29.6	0.8	8.00E-06	27.3	0.1	2.4	1	**FUN_034356-T1**	FUN_034356
120	**1.90E-06**	29.3	1.7	0.052	15.1	0.1	2.5	2	**FUN_031855-T1**	FUN_031855
121	**2.70E-06**	28.9	0.1	5.40E-06	27.9	0.1	1.6	2	**FUN_005998-T1**	FUN_005998
122	**5.90E-06**	27.8	0	9.80E-06	27.1	0	1.4	1	**FUN_022861-T1**	FUN_022861
123	**1.10E-05**	26.9	0.1	2.10E-05	26	0.1	1.5	1	**FUN_032145-T1**	FUN_032145
124	**1.20E-05**	26.8	0	0.05	15.2	0	2.7	2	**FUN_022741-T1**	FUN_022741
125	**2.90E-05**	25.5	0.3	0.0002	22.9	0.2	2.3	1	**FUN_026291-T1**	FUN_026291
126	**3.00E-05**	25.5	0.1	0.0001	23.8	0.1	1.9	1	**FUN_028364-T2**	FUN_028364
127	**5.50E-05**	24.7	0.3	0.00092	20.7	0.1	2.1	2	**FUN_000539-T1**	FUN_000539
128	**6.80E-05**	24.4	0	6.80E-05	24.4	0	3.4	1	**FUN_039841-T1**	FUN_039841
129	**7.30E-05**	24.3	0	0.3	12.7	0	2.6	2	**FUN_031151-T1**	FUN_031151

130	**0.00014**	23.3	3.4	0.013	17.1	0.4	2.2	2	**FUN_039075-T1**	FUN_039075
131	**0.00017**	23.1	0.8	0.0033	19	0.2	2.4	2	**FUN_029798-T1**	FUN_029798
132	**0.00024**	22.6	0	0.053	15.1	0	1.9	2	**FUN_031903-T1**	FUN_031903
133	**0.00024**	22.6	0.2	0.00038	22	0.2	1.3	1	**FUN_007020-T1**	FUN_007020
134	**0.00034**	22.1	0.2	0.0026	19.3	0.1	2.6	1	**FUN_005282-T1**	FUN_005282
135	**0.00065**	21.2	0	0.0012	20.4	0	1.5	1	**FUN_011654-T1**	FUN_011654
136	**0.00079**	21	0.3	0.0012	20.4	0.1	1.5	1	**FUN_000548-T1**	FUN_000548
137	**0.00089**	20.8	0	0.015	16.9	0.2	2.5	2	**FUN_011970-T1**	FUN_011970
138	**0.0009**	20.8	0.1	0.0027	19.2	0.1	1.8	1	**FUN_016917-T1**	FUN_016917
139	**0.0011**	20.5	1.4	0.0031	19.1	0.2	2.2	1	**FUN_000546-T1**	FUN_000546
140	**0.0014**	20.2	1	0.0078	17.8	0.9	2.4	1	**FUN_024126-T1**	FUN_024126
141	**0.0016**	20	0	0.78	11.4	0	2.6	2	**FUN_034657-T2**	FUN_034657
142	**0.0018**	19.8	0.5	0.0068	18	0.5	1.9	2	**FUN_010442-T1**	FUN_010442
143	**0.0043**	18.6	0	0.0079	17.8	0	1.5	1	**FUN_002455-T1**	FUN_002455
144	**0.0044**	18.6	0	0.2	13.3	0	2.9	2	**FUN_038424-T1**	FUN_038424
145	**0.0048**	18.4	0.6	0.024	16.2	0.3	2.4	1	**FUN_004232-T1**	FUN_004232
146	**0.0056**	18.2	3.3	0.1	14.2	0.3	2.4	2	**FUN_037993-T2**	FUN_037993
147	**0.0062**	18.1	0.5	0.014	17	0.3	1.8	1	**FUN_031856-T1**	FUN_031856
148	**0.0064**	18	0	0.015	16.9	0	1.6	1	**FUN_038743-T2**	FUN_038743
149	**0.0068**	18	0	0.012	17.2	0	1.4	1	**FUN_018032-T1**	FUN_018032
150	**0.0074**	17.8	0.3	0.018	16.6	0.3	1.7	1	**FUN_016918-T1**	FUN_016918
**inclusion threshold**										
	0.016	16.8	2	0.15	13.6	0.3	2.4	2	FUN_037993-T4	FUN_037993
	0.017	16.7	0.9	0.047	15.3	0.9	1.8	1	FUN_034049-T1	FUN_034049
	0.024	16.2	2	0.18	13.4	0.3	2.3	2	FUN_037993-T3	FUN_037993
	0.031	15.9	2	0.21	13.2	0.3	2.4	2	FUN_037993-T1	FUN_037993
	0.032	15.8	0.1	0.092	14.3	0.1	1.7	1	FUN_015531-T1	FUN_015531
	0.038	15.6	0.1	0.61	11.7	0	2.5	2	FUN_031560-T1	FUN_031560
	0.04	15.5	0	0.11	14	0	1.8	1	FUN_013386-T1	FUN_013386
	0.06	14.9	0	1.7	10.3	0	2.8	2	FUN_013033-T1	FUN_013033
	0.081	14.5	0	0.22	13.1	0	1.8	1	FUN_013070-T1	FUN_013070
	0.097	14.3	0	3.8	9.2	0	3	2	FUN_029615-T1	FUN_029615
	0.15	13.7	0	1.9	10.1	0	2.5	2	FUN_036972-T1	FUN_036972
	0.15	13.6	0.1	7.9	8.1	0	2.3	1	FUN_032392-T1	FUN_032392
	0.16	13.5	0.1	0.6	11.7	0	1.9	1	FUN_014368-T1	FUN_014368
	0.22	13.1	0	0.51	11.9	0	1.7	1	FUN_004403-T1	FUN_004403
	0.26	12.9	0.2	1.6	10.3	0.1	2.3	1	FUN_030760-T1	FUN_030760
	0.26	12.9	0	0.76	11.4	0	1.8	1	FUN_011778-T1	FUN_011778
